# Functional amyloids as inhibitors of plasmid DNA replication

**DOI:** 10.1038/srep25425

**Published:** 2016-05-05

**Authors:** Laura Molina-García, Fátima Gasset-Rosa, María Moreno-del Álamo, M. Elena Fernández-Tresguerres, Susana Moreno-Díaz de la Espina, Rudi Lurz, Rafael Giraldo

**Affiliations:** 1 Department of Cellular and Molecular Biology, Centro de Investigaciones Biológicas – CSIC, E28040 Madrid, Spain; 2 Max Planck Institute for Molecular Genetics, D14195 Berlin, Germany

## Abstract

DNA replication is tightly regulated to constrain the genetic material within strict spatiotemporal boundaries and copy numbers. Bacterial plasmids are autonomously replicating DNA molecules of much clinical, environmental and biotechnological interest. A mechanism used by plasmids to prevent over-replication is ‘handcuffing’, i.e. inactivating the replication origins in two DNA molecules by holding them together through a bridge built by a plasmid-encoded initiator protein (Rep). Besides being involved in handcuffing, the WH1 domain in the RepA protein assembles as amyloid fibres upon binding to DNA *in vitro*. The amyloid state in proteins is linked to specific human diseases, but determines selectable and epigenetically transmissible phenotypes in microorganisms. Here we have explored the connection between handcuffing and amyloidogenesis of full-length RepA. Using a monoclonal antibody specific for an amyloidogenic conformation of RepA-WH1, we have found that the handcuffed RepA assemblies, either reconstructed *in vitro* or in plasmids clustering at the bacterial nucleoid, are amyloidogenic. The replication-inhibitory RepA handcuff assembly is, to our knowledge, the first protein amyloid directly dealing with DNA. Built on an amyloid scaffold, bacterial plasmid handcuffs can bring a novel molecular solution to the universal problem of keeping control on DNA replication initiation.

The molecular mechanisms of DNA replication in Gram-negative bacteria have been the subject of intense research for four decades. In the case of most plasmids, a plasmid-encoded protein (Rep) triggers replication in a regulated way^1^. In RepA from the *Pseudomonas* pPS10 plasmid replicon^2^, its N-terminal *winged-helix* dimerization domain (WH1) is structurally remodelled upon binding to DNA, resulting in the transformation of stable transcriptional repressor dimers into metastable replication-competent monomers^3^. In the plasmid replication origin (*oriV*) RepA monomers assemble the initiation complex at specific directly repeated sequences (iterons)^4^,^5^. Once replicated, two iteron-containing plasmid DNA molecules get coupled in a ‘handcuffed’ complex through interactions mediated by origin-bound Rep molecules, sterically hindering premature replication rounds^6^,^7^,^8^,^9^,^10^. In previous work, we characterized that the handcuffed complexes responsible for negative regulation of pPS10 replication were mediated by RepA monomers coupled through their WH1 domains, albeit involving a distinct interface to that found in the repressor RepA dimers^11^.

Tracking the molecular basis of the intrinsic tendency of RepA towards aggregation, we found that monomers of the isolated RepA-WH1 domain assemble into amyloid fibres *in vitro*^12^,^13^. A plasmid-specific dsDNA sequence acts as allosteric effector of amyloidosis^12^,^14^. When fused to a fluorescent protein marker, RepA-WH1 behaves as a proteinopathic, vertically transmissible (from mother cell to daughter cells) prionoid in *Escherichia coli* thus enabling bacteria as a model system for approaching protein amyloidosis^15^,^16^. We have recently described a monoclonal antibody (B3h7) specific for an oligomeric conformation of RepA-WH1 on pathway towards building amyloid fibres^17^. B3h7 thus overcame limitations imposed by the poor reactivity of RepA-WH1 towards commercially available anti-amyloid antibodies (such as A11 and OC)^17^. Using B3h7, we discovered that pre-amyloidogenic RepA-WH1 oligomers assemble at the bacterial nucleoid^17^, as expected from the DNA-promoted amyloidogenesis of the protein *in vitro*^12^,^14^.

Protein amyloids, in their fibrillar or oligomeric aggregated states, are infamous as the causative agents of human degenerative proteinopathies spanning from Alzheimer’s, Parkinson’s, Huntington’s, prion diseases and amyotrophic lateral sclerosis to dialysis-related amyloidosis and type-II diabetes^18^,^19^. However, work performed on yeast prions^20^ and bacterial biofilms^21^ have clearly shown that amyloids can also be functional, i.e. provide microorganisms with quickly selectable epigenetic, gain of function phenotypes^22^. Here we explore the link between regulation of pPS10 replication by RepA-mediated origin handcuffing and DNA-promoted RepA-WH1 amyloidosis. We have found that the regulatory RepA handcuffs actually are, to our notice, the first intracellular functional amyloids found in bacteria or involved in DNA replication.

## Results

### Amyloidogenic RepA mediates handcuffed plasmid complexes *in vitro*

An important open question is whether the basis of two phenomena involving the WH1 domain, i.e. handcuffing of RepA-iteron complexes and amyloidosis of RepA-WH1, were or not a single process (amyloidogenesis), albeit in naturally functional or synthetic proteinopathic contexts, respectively. With such purpose, we have used B3h7, a monoclonal antibody specific for on-pathway amyloidogenic RepA-WH1 oligomers^17^, as a probe to explore a possible contribution of amyloidogenesis to handcuffing.

**Figure 1:**
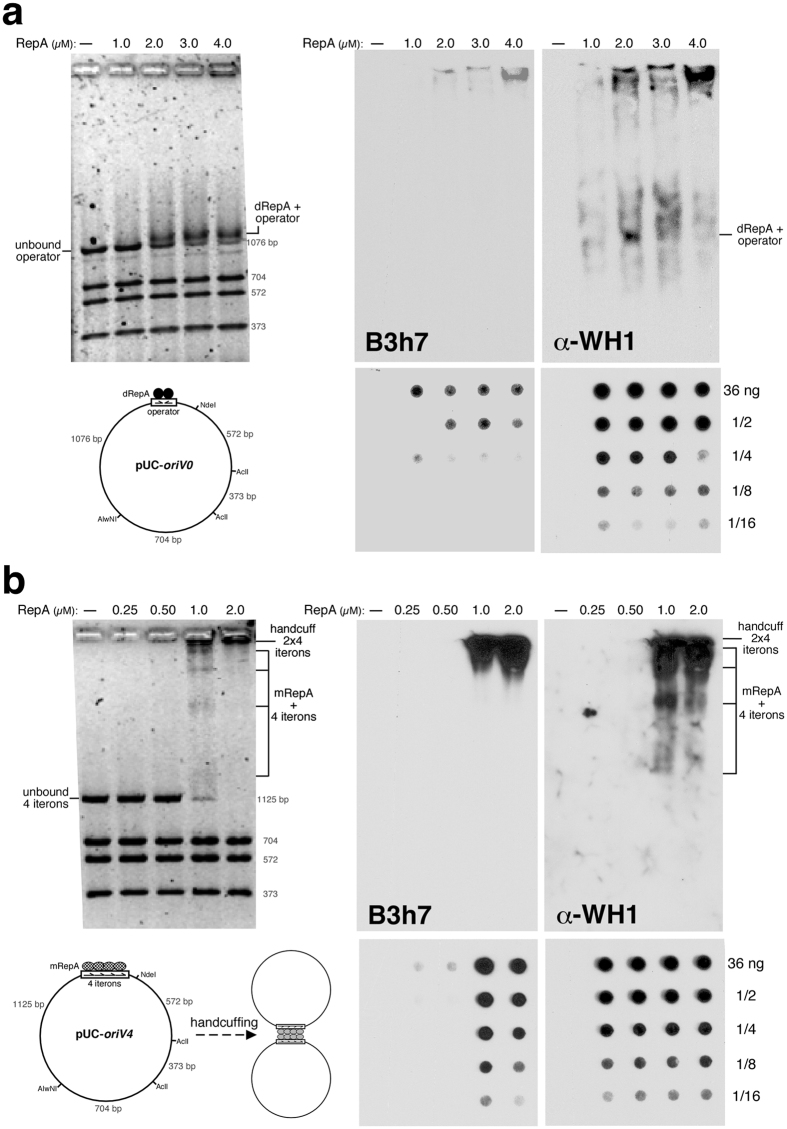
Biochemical test of the amyloidogenicity of RepA-DNA complexes. Antibodies used recognize RepA-WH1 either irrespective of its conformation (polyclonal α-WH1) or as amyloidogenic oligomers (monoclonal B3h7)^17^. Complexes assembled *in vitro* between full-length RepA and ≈1 kb plasmid restriction fragments carrying either the *repA* operator (pUC-*oriV0*) (**a**) or the four pPS10 iterons (pUC-*oriV4*) (**b**), were resolved in agarose gels (*left*) and then Western-blotted to membranes before incubation with the indicated antibodies (*right*). Duplicates of the same samples were serially diluted and then directly dot-blotted to membranes before incubation with the same antibodies (*bottom*). Plasmid schemes highlight the distinct RepA conformations involved in transcriptional repression (black circles), DNA replication initiation (chequered ovals) and handcuffing (grey ovals)^3^,^4^,^5^,^11^. B3h7 preferentially recognizes the high molecular weight RepA-*oriV* complexes at the conditions in which two origin fragments appeared handcuffed in *trans*^11^ (Fig. 2, *right*), pointing to an amyloidogenic nature for such regulatory complex.

**Figure 2:**
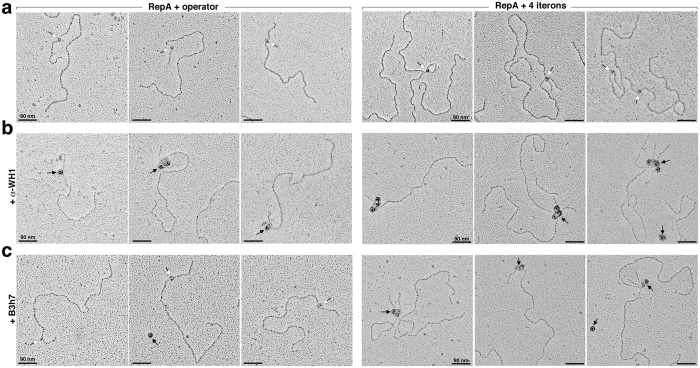
iEM visualization of amyloidogenic oligomers of RepA bridging origin-coupled plasmid molecules. Image galleries of *in vitro* reconstituted complexes between full length RepA and plasmids carrying the pPS10 *repA* operator (*left*) or the four iterons array at *oriV* (*right*). Linearized plasmid molecules were incubated with RepA (**a–c**) and antibodies (**b,c**) before negative staining and Pt-shadowing. (**a**) Besides free plasmid molecules, oligomeric RepA-DNA complexes are evident (white arrows) for both types of sequences. (**b**) iEM with the primary polyclonal α-WH1 antibody and a secondary gold-conjugated antibody (Au-IgG, 10 nm Ø; black arrows). RepA is found at the core of the assemblies identified in (**a**), either as bound to the operator (*left*) or as origin-bound or handcuffed (*right*). (**c**) iEM with the monoclonal anti-oligomeric amyloid antibody B3h7 and secondary Au-IgG (black arrows). B3h7 does not efficiently recognize the operator-bound RepA dimers (white arrows; the single black arrow marks a background antibody molecule) (*left*), whereas B3h7 identifies (black arrows) the oligomers assembled by the RepA monomers at the handcuffed complexes as amyloidogenic (*right*).

We reconstructed *in vitro* the complexes between full-length RepA and plasmid DNA molecules carrying the pPS10 operator or iteron sequences^11^ and then performed Western/dot-blotting (Fig. 1) or immuno-electron microscopy (iEM) (Fig. 2) using the B3h7 antibody. α-WH1, a polyclonal antibody specific for RepA-WH1 but not its conformation^17^, was also tested in these assays as a control. We thus surveyed if RepA adopts an amyloid structure in two distinct functional assemblies: i) RepA dimers bound at the *repA* operator inverted repeat; and ii) RepA monomers titrated on the *oriV* iterons, either as handcuffed complexes or as the intermediates of binding.

The titration of RepA on operator (Fig. 1a) and *oriV* iteron (Fig. 1b) DNA sequences, present in plasmids that had been sliced into pieces through multiple restriction digestion, showed specific mobility shifts (native EMSA) only for the fragment carrying the relevant pPS10 sequences. In the case of RepA binding to the iterons, Western blotting with the B3h7 antibody revealed an intense hybridization signal solely for the highest molecular weight complex, located close to the well of the gel, but not for any of the intermediate monomers binding cooperatively^4^ to the four iterons at *oriV* (Fig. 1b). On the contrary, the signal generated at the well for the samples including the operator was significantly less intense than that observed for the iterons, i.e. some protein aggregation happened but no signal showed up for the specific complex between RepA dimers and DNA (Fig. 1a). The control α-WH1 antibody recognized every complex in which a RepA molecule was taking part, either as a dimer at *repA* operator (Fig. 1a) or as the individual monomers binding to each iteron (Fig. 1b). Dot-blot analysis of serial dilutions of the titration points for both types of DNA sequences revealed that samples including RepA-iteron complexes (Fig. 1b) were labelled with B3h7 up to higher dilutions than those with RepA-operator complexes (Fig. 1a) and, importantly, only at the titration points in which handcuffing complexes were evident in EMSA, whereas α-WH1 recognized both kinds of samples to a similar extent. The differences observed between the hybridization patterns for both antibodies speak to their distinct specificities, as recently reported^17^: either for an oligomeric and amyloidogenic conformation of RepA (B3h7) or for multiple peptide epitopes distributed across RepA regardless the conformation or association state of the protein (α-WH1). In summary, these approaches indicate that the largest complexes built by RepA at the *oriV* iterons, corresponding to handcuffed origin molecules^11^, involve amyloidogenic oligomers, whereas the individual RepA monomer-iteron and RepA dimer-operator complexes do not.

**Figure 3:**
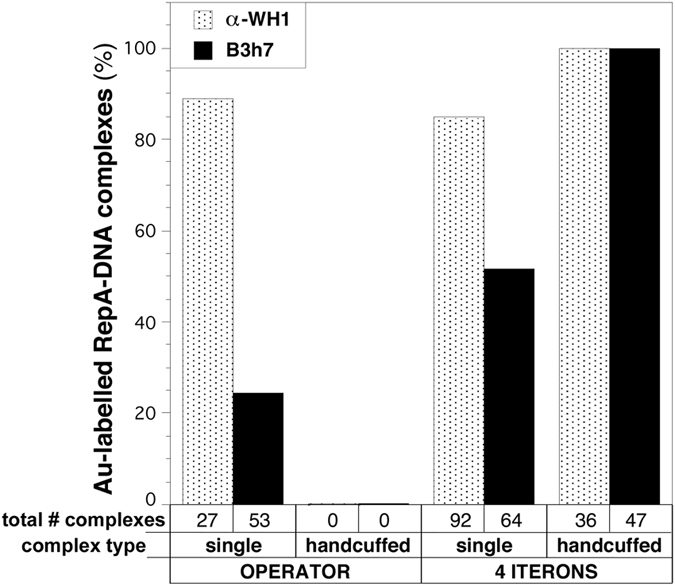
Quantitative analysis of the reconstituted, and iEM-visualized, complexes assembled by RepA at plasmids carrying operator or iteron sequences. Particles were classified according to the assembly type of the DNA molecules (single/linear *vs*. double/handcuffed) and to the distinct reactivity of RepA towards the α-WH1 and B3h7 antibodies (see Fig. 2). RepA complexes with the iteron tandem repeats, with a preference for the handcuffed state, were identified as amyloidogenic by the B3h7 probe. Data were compiled from three independent reconstitutions plus EM-imaging.

Complementary iEM analysis of the individual RepA-DNA complexes, as reconstituted on linearized plasmid molecules (Fig. 2a), indicated that while the α-WH1 polyclonal antibody recognized any RepA particle bound to DNA, either RepA dimers at the operator (Fig. 2b, *left*) or RepA monomers at the iterons (Fig. 2b, *right*), labelling by the amyloidogenic oligomer-specific monoclonal B3h7 antibody (Fig. 2c) showed a clear preference for the latter (Fig. 2c, *right*). A quantitative analysis of the iEM images revealed that B3h7 labelled all the handcuffed complexes, but it also recognized half of the single uncoupled RepA-*oriV* particles (Fig. 3). Whether the latter were from dissociation of handcuffed complexes during handling of the samples cannot be excluded. In contrast, and compatible with the findings in the Western and dot-blot assays (Fig. 1), B3h7 barely labelled one-fourth of the RepA-operator complexes (Fig. 3). By focusing at a nanometer scale on individual complexes, iEM with the B3h7 probe provides unambiguous evidence for the existence of an amyloidogenic structure for the bridge made of RepA that holds handcuffed plasmids together (see scheme in Fig. 1b).

### Amyloidogenic RepA handcuffing at the nucleoid of *P. aeruginosa* cells carrying pPS10

**Figure 4:**
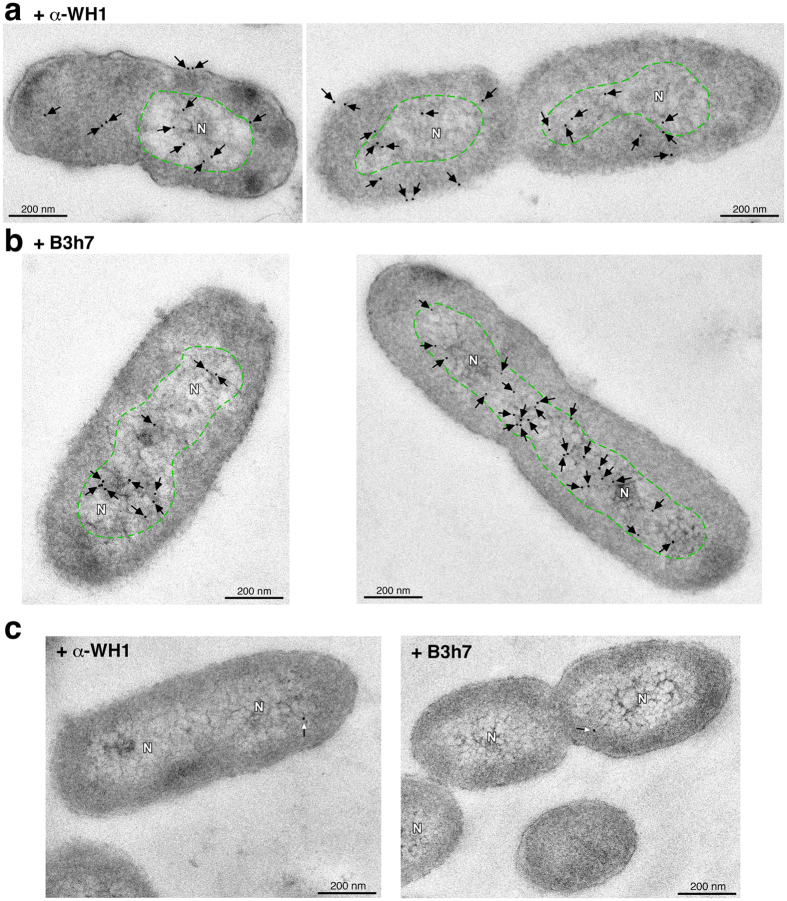
Amyloidogenic (handcuffed) RepA at the nucleoid of proliferating *P. aeruginosa* cells carrying pPS10. (**a**) iEM performed with the α-WH1 polyclonal antibody on ultra-thin sections (≈90 nm) of *P. aeruginosa* PAO1024 cells bearing a functional pPS10 plasmid replicon. RepA molecules (arrows pointing to Au particles) appeared sparsely distributed across the whole bacterial cells. (**b**) iEM with the anti-amyloid B3h7 antibody probe on cells from the same bacterial cultures shown in (**a**). Amyloidogenic RepA oligomers (arrows) were located as clusters inside the nucleoid (N, green sectors), resembling the amyloid precursors of the RepA-WH1 prionoid previously identified at the *E. coli* nucleoid^17^. (**c**) Negative control: plasmid-free bacterial cells probed with the α-WH1 and B3h7 antibodies; *arrows*, background Au particles.

The B3h7 antibody combined with the nanometer-range resolution of electron microscopy were successful in detecting foci of the amyloidogenic RepA-WH1 prionoid at the nucleoid of *E. coli* cells^17^. Therefore, the same approach was also the choice for imaging the whole RepA protein, not just its N-terminal WH1 domain, as expressed from its natural promoter in a pPS10 replicon undergoing controlled RepA-dependent DNA replication in *P. aeruginosa* cells, the natural host for the plasmid (Fig. 4). As for the aggregated amyloid precursors of the RepA-WH1 prionoid in *E. coli* cells^17^, in *P. aeruginosa* B3h7 labelled the nucleoid territory (Fig. 4b), whereas signal from the polyclonal α-WH1 antibody, besides the nucleoid, extended also to the cytoplasm probably pointing to RepA molecules unbound to DNA (Fig. 4a). As expected from their high specificity for RepA, none of the two antibodies targeted bacteria not carrying a pPS10 replicon (Fig. 4c). Labelling by B3h7 of both the nucleoid of bacteria carrying pPS10 (Fig. 4b) and the handcuffs reconstituted *in vitro* (Figs 1b and 2c, *right*) provide strong support to the proposal that the regulatory nucleoprotein complexes involved in the control of plasmid DNA replication are built on an amyloidogenic RepA backbone.

## Discussion

**Figure 5:**
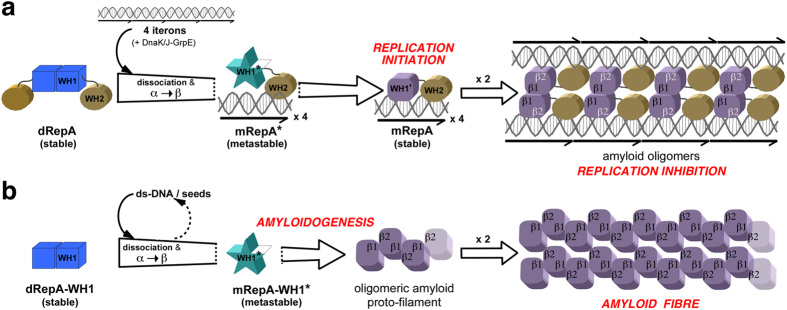
Schematic models of the amyloid assemblies built by RepA. (**a**) RepA in negative control of DNA replication (handcuffing)^11^. (**b**) RepA-WH1 in the polymerization of prionoid fibres^13^. Starting with soluble and stable dimers, dsDNA-promoted amyloidogenesis would drive the WH1 domain, in both scenarios and through aggregation-prone metastable protein monomers, into two distinct amyloid structures: WH1 domains paired *in trans* through a head-to-head interface (β1-β1), resulting in inhibition of DNA replication (**a**)^11^, or head-to-tail interactions involving a second, yet undefined interface (β1–β2), which build fibres with indefinite length (**b**)^17^. The factor limiting the span of RepA complexes in handcuffing (**a**) is the specific binding of the protein to the four iteron DNA direct repeats, as determined by the C-terminal WH2 domain^3^,^4^,^5^ that both stabilizes the nucleoprotein complex and shields the second amyloidogenic interface (β2, in white) avoiding subsequent polymerization.

The findings reported here integrate amyloids in the complex conformational transactions experienced by RepA protein in a key process for the biology of plasmid extrachromosomal mobile genetic elements in Gram-negative bacteria: i) DNA (operator)-bound dimers repress *repA* gene transcription; ii) DNA (iteron)-bound monomers initiate DNA replication; and iii) by means of handcuffing, amyloidogenic oligomers inhibit post-replicative origin firing. A previous study^11^ established that handcuffing complexes involve RepA monomers coupled as pseudo-dimers through a surface in WH1 that includes the C-terminus of helix α2, which is distinct from the dimerization interface found in soluble and operator-bound RepA dimers^3^. Since the key amyloidogenic stretch in RepA-WH1 is also found at the C-terminus of α2^12^, the findings reported here are fully compatible with a functional amyloid being involved in the control of pPS10 replication (Fig. 5). An amyloid protein core confers a high stability to the handcuffed complexes, providing a basis for the known requirement of the DnaK-DnaJ-GrpE chaperone triad in disassembling Rep handcuffs to allow for further plasmid replication rounds^8^,^10^. Interestingly, DnaK modulates the amyloidogenesis of the RepA-WH1 prionoid *in vivo* by shifting the equilibrium between an acutely and a mildly cytotoxic transmissible conformations (strains) of the protein towards the latter^16^.

The understanding of plasmid partition^23^, i.e. the molecular mechanism assuring the equal distribution of plasmid copies between daughter cells upon division, has recently advanced in a substantial way^24^,^25^. Partition of low copy-number plasmids relies on centromere-like sequences (*parS*), a *parS*-binding protein (ParB) and an ATP hydrolysing protein (ParA) which couples the partition complex to the nucleoid, then spread the plasmids to distal locations through diffusion ratchet^26^ or DNA looping^27^ mechanisms. In all cases studied so far, interactions involving ParA and ParB are mediated through α-helical interfaces^24^,^25^, not by β-sheets, as proposed here for RepA handcuffing. It is likely that handcuffing of plasmids is the basis for the amyloidogenic patches found in the nucleoid of bacteria carrying pPS10 (Fig. 3b). Plasmids lacking its own partition module but carrying sequences that match the centromere-like *parS* locus still become associated to the bacterial nucleoid for stable segregation by the ParAB proteins encoded at the chromosome^28^. This is likely the case for pPS10, because its replicon includes sequences (e.g.: C_521_CTTCCATGGGGAAGG_536_)^2^ bearing similarity to the consensus *parS* in *P. aeruginosa*^29^. Beyond its role in replication control, whether amyloid-mediated handcuffing has any effect on plasmid partition deserves further exploration, since the stable Rep-iteron complexes have been recently found to constrain ParB spreading^30^. Although speculative, it would be sufficient that at least one of the plasmids bridged through handcuffing would stay attached through its *parS* sites to the chromosome to achieve efficient partition of a plasmid cluster.

CPEB protein leads the list of functional amyloids dealing with mRNA, contributing to establish memory in neurons^30^,^31^. Analogously, the handcuffed RepA oligomer might be the foundational member of a class of amyloids modulating the dynamics of DNA and genomes.

## Methods

### Electrophoretic mobility-shift assays (EMSA)

Plasmids pUC-*oriV0* (*repA* operator) and pUC-*oriV4* (pPS10 four iterons)^11^ were digested with NdeI, AclI and AlwNI, to generate four fragments of which the largest one (≈ 1 kb) included the relevant sequence probes (see Fig. 1), and then purified through the FastPlasmid Mini Kit (5Prime, Fisher). Concentrations of the DNA probes were determined after gel electrophoresis, using calibrated MW standards and the Quantity-One software (Bio-Rad). Each binding reaction included 37 ng of the relevant dsDNA probe, acting the other three fragments in the incubation mixture as controls for non-specific binding. Purified histidine-tagged RepA-WT^11^ was pre-treated with 1 M urea at 4 °C for 15 min, to assure a monodisperse dimeric state for the protein, and then titrated on the dsDNA probes in a final volume of 10 μL (binding buffer: 0.05 M NaCl, 0.025 M Hepes pH 8, 5% glycerol, 0.1 mg mL^−1^ BSA). After 15 min on ice, samples were loaded in 1.5% agarose-0.25xTBE gels and electrophoresis performed at 100 V for 2 h at 4 °C. Gels were stained with Gel-Red (Biotium) and fluorescence emission images captured using a Gel-Doc 2000 (Bio-Rad).

### Western and dot-blot assays

α-WH1 and B3h7 antibodies were obtained and purified as previously described^17^. Western blot assays were performed after EMSA by transferring the agarose gels to PVDF membranes in a Trans-Blot cell (Bio-Rad) at 16 V (400 mA), 4 °C for 15 h in 1 × TAE buffer supplemented with 0.025% SDS. For dot-blots, nitrocellulose membranes (0.45 μm ø pore; Bio-Rad) were set in a Bio-Dot microfiltration device (Bio-Rad). Wells were pre-equilibrated twice by flowing 200 μL of binding buffer (no BSA added). 72 ng of RepA in complex with DNA, replicas of the samples assembled for EMSA (see above), were serially diluted (2-fold steps) in the same buffer (200 μl final volume) and immediately spotted under gravity flow. In both assays, blotted membranes were then blocked, for 1 h at room temperature, with 5% skimmed powder milk in Tris-buffered saline buffer (pH 7.0) containing 0.01% Tween-20 (TBS-T) and probed for 2 h at room temperature with the primary antibodies (B3h7 1:3,000; or α-WH1 1:1,000) in TBS-T. The membranes were then washed three times with TBS-T and incubated for 1 h with the appropriate (mouse or rabbit, respectively) HRP-conjugated secondary antibodies (1:10,000). After three additional washes with TBS-T, chemiluminiscent detection was performed on X-ray films with the ECL Prime kit (GE Healthcare).

### Electron microscopy

*TEM/iEM of in vitro assembled RepA-DNA complexes*: 40 ng of histidine-tagged RepA-WT were added to 40 ng of NdeI-linearized pUC-*oriV0* or pUC-*oriV4* (see above)^11^ in 10 μl of 200 mM KCl, 7 mM Mes (pH 6.0), 1.5 mM DTT, 3% glycerol, 1 mM ATP and incubated for 10 min at 4 °C. Glutaraldehyde was then supplied to 0.2% and cross-linking reaction proceeded for 10 min before quenching, by adding 2 μl of 0.5 M glycyl-glycine (pH 7.4) for 10 extra min. The reaction mixes were incubated with the primary antibodies (α-WH1 or B3h7, 0.6 ng.μl^−1^) for 30 min at room temperature and then diluted to 40 μl with TBT (100 mM Tris-HCl pH 7.5, 100 mM NaCl, 10 mM MgCl_2_). Samples were gel-filtrated through a TBT-Sephacryl S-500 column (GE Healthcare) and peak fractions were pooled and clarified by centrifugation (800× g, for 5 min). The Au-conjugated secondary antibodies (either anti-mouse or anti-rabbit, 1:40) were incubated with the samples for 30 min at room temperature. Reactions were further processed to clean unbound antibodies through the gel-filtration column as above, but equilibrated in 10 mM triethanolamine-HCl pH 7.5, 10 mM MgCl_2_. Samples were sequentially adsorbed on mica, stained with 2% uranyl acetate, platinum shadowed and carbon coated, before examination of the specimens in a FEI CM100 electron microscope, as described^11^.

*iEM of bacterial cells*: Cultures of *P. aeruginosa* PAO1024 carrying pRG14, a pPS10 replicon derivative encoding *repA* and *oriV* 2, were harvested in late exponential phase (OD_600_ = 1.0). Cells were fixed, embedded in resin, sliced, sequentially incubated on the grids with the primary antibodies (0.2 ng μl^−1^) and the secondary Au-conjugated antibodies and stained with uranyl acetate, as described in^15^,^16^,^17^. Specimens were examined in a JEOL JEM-1230 electron microscope, as indicated^15^,^16^,^17^.

## Additional Information

**How to cite this article**: Molina-García, L. *et al*. Functional amyloids as inhibitors of plasmid DNA replication. *Sci. Rep*. **6**, 25425; doi: 10.1038/srep25425 (2016).
